# Targeting mutant p53 for cancer therapy: direct and indirect strategies

**DOI:** 10.1186/s13045-021-01169-0

**Published:** 2021-09-28

**Authors:** Jiahao Hu, Jiasheng Cao, Win Topatana, Sarun Juengpanich, Shijie Li, Bin Zhang, Jiliang Shen, Liuxin Cai, Xiujun Cai, Mingyu Chen

**Affiliations:** 1 Department of General Surgery, Sir Run-Run Shaw Hospital, Zhejiang University, No. 3 East Qingchun Road, Hangzhou, 310016 China; 2 School of Medicine, Zhejiang University, Hangzhou, 310058 China; 3 Engineering Research Center of Cognitive Healthcare of Zhejiang Province, Zhejiang Province, Hangzhou, China; 4 Key Laboratory of Endoscopic Technique Research of Zhejiang Province, No. 3 East Qingchun Road, Hangzhou, 310016 China

**Keywords:** p53, Cancer therapy, MDM2 inhibitor, p53 restoration, Synthetic lethality, Noncoding RNA

## Abstract

TP53 is a critical tumor-suppressor gene that is mutated in more than half of all human cancers. Mutations in *TP53* not only impair its antitumor activity, but also confer mutant p53 protein oncogenic properties. The p53-targeted therapy approach began with the identification of compounds capable of restoring/reactivating wild-type p53 functions or eliminating mutant p53. Treatments that directly target mutant p53 are extremely structure and drug-species-dependent. Due to the mutation of wild-type p53, multiple survival pathways that are normally maintained by wild-type p53 are disrupted, necessitating the activation of compensatory genes or pathways to promote cancer cell survival. Additionally, because the oncogenic functions of mutant p53 contribute to cancer proliferation and metastasis, targeting the signaling pathways altered by p53 mutation appears to be an attractive strategy. Synthetic lethality implies that while disruption of either gene alone is permissible among two genes with synthetic lethal interactions, complete disruption of both genes results in cell death. Thus, rather than directly targeting p53, exploiting mutant p53 synthetic lethal genes may provide additional therapeutic benefits. Additionally, research progress on the functions of noncoding RNAs has made it clear that disrupting noncoding RNA networks has a favorable antitumor effect, supporting the hypothesis that targeting noncoding RNAs may have potential synthetic lethal effects in cancers with p53 mutations. The purpose of this review is to discuss treatments for cancers with mutant p53 that focus on directly targeting mutant p53, restoring wild-type functions, and exploiting synthetic lethal interactions with mutant p53. Additionally, the possibility of noncoding RNAs acting as synthetic lethal targets for mutant p53 will be discussed.

## Introduction

**Fig. 1:**
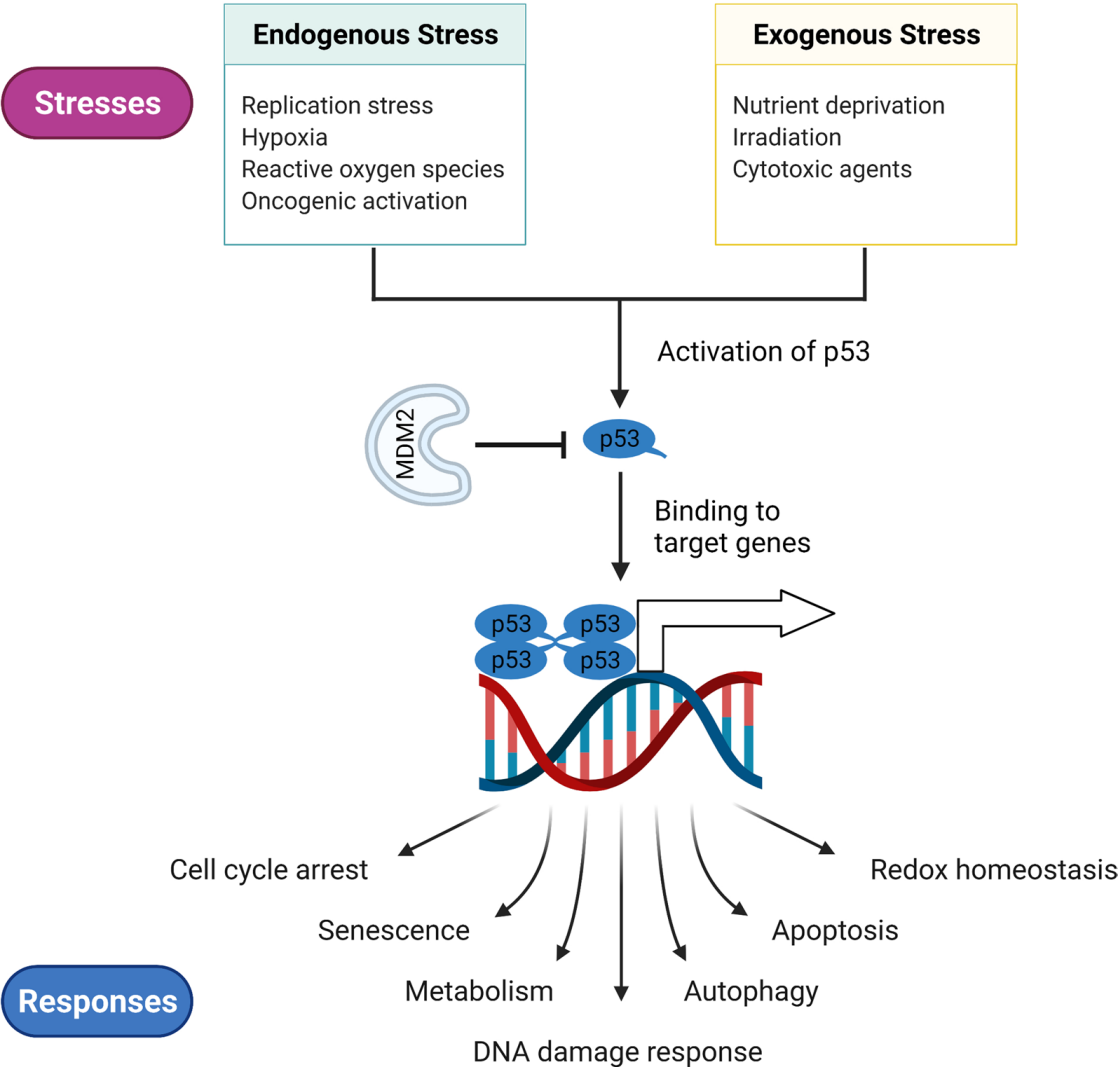
The functions of p53 in the normal cells. p53 is an important tumor suppressor in normal cells to maintain homeostasis. Throughout their lifespan, cells are faced with continuing stresses including endogenous and exogenous stresses. To overcome these stresses, p53 is activated to mediate a series of cellular responses via its transcription-dependent functions or direct protein-to-protein interactions. p53-mediated responses also rely on the type and degree of insults, as well as the cell types and the context in which the insult occurs

The tumor-suppressor p53, encoded by the *TP53* gene (or *Trp53* in mice), is critical for normal cell growth and tumor prevention [1, 2]. Generally, the p53 protein is kept at a low level in normal tissue by its negative regulator, mouse double minute 2/X (MDM2/X) [3]. Numerous endogenous and exogenous stressors can activate p53, triggering it to further regulate a series of cellular responses necessary for homeostasis maintenance (Fig. 1) [4]. The activation of p53 in response to multiple stresses is critical for normal cells to survive and protect themselves from tumorigenesis. However, TP53 is frequently mutated in most human cancers, resulting in the loss of functions (LOFs) necessary for tumor suppression and even the gain of functions (GOFs) necessary for tumor growth [5, 6]. The most common p53 mutation is the missense mutation in the DNA-binding domain (DBD), which affects only one amino acid in the p53 protein but has a significant effect on the protein's function [7]. Tumors harbor p53 mutations frequently progress more rapidly, have a poor response to anticancer therapy, and have a poor prognosis [6, 8, 9]. Therefore, targeting p53 for cancer therapy is an attractive strategy.

Depending on the p53 status, p53 treatments may include preventing the degradation of wild-type p53 (wtp53), suppressing mutant p53 (mutp53), or restoring the wild-type functions of mutp53 [10, 11]. Agents that protect wtp53 primarily act by interfering with the interactions of p53 and its negative regulators, most notably MDM2, to prevent subsequent ubiquitination [12]. Increased wtp53 levels are sufficient to induce tumor-suppressive responses [13]. Different strategies for restoring p53 functions have been developed based on the variety of mutp53 protein structures as well as their specific functional deficiencies [14]. Additionally, p53 GOF mutations confer oncogenic properties on cancer cells, and thus, targeting these specific mutations may inhibit cancer cell proliferation [15]. Despite their relative advantages, these treatments have a very limited effect due to the prevalence of mutp53 variants. Thus, a superior strategy that specifically targets the majority of mutant p53 can result in greater therapeutic benefit for patients.

Since the successful use of the PARP1 inhibitor olaparib in the treatment of cancers caused by *BRCA1* and *BRCA2* mutations, such as breast, ovarian, pancreatic, and prostate cancers, synthetic lethality-based anticancer therapy has garnered enormous attention [16]. The term "synthetic lethality" was originated in Drosophila research and refers to the fact that disrupting partial genes via synthetic lethal interactions is feasible but lethal [17, 18]. As a result, exploiting and targeting synthetic lethal partners may become an attractive therapeutic strategy for unmodifiable genes. *TP53* is an example of an “undruggable” gene that frequently loses its normal functions and activates a cascade of signaling pathways to promote tumor progression and compensate for the loss of functions. Numerous synthetic lethality partners may be concealed within these altered pathways. As one of the most prominent functions of p53, it is not surprising that researchers screen for p53 synthetic lethal partners associated with the cell cycle. Mutp53-positive cancers lose their capacity to induce G1 arrest, which is maintained by wtp53 and its transcriptionally activated p21 [19]. Following DNA damage, cancer cells harboring mutp53 are protected from accumulated replication stress (RS) and mitotic catastrophe by intra-S and G2 arrest, which are regulated by multiple factors [20, 21]. The G2 checkpoint contains the first identified synthetic lethal interaction with p53-deficient cancer cells [22]. Tumors lacking p53 are particularly susceptible to G2 checkpoint abrogation when exposed to DNA damage. Certain regulators of the S and G2 arrest have been shown to be synthetic lethal to p53. However, researchers are intrigued by alternative p53-mediated pathways that have a synthetic lethal effect when combined with mutp53. Additionally, synthetic lethality is a genetic concept, and its realization ultimately requires the disruption of protein functions. With the growing body of knowledge about the functions of noncoding RNAs (ncRNAs), we can assume that a synthetic lethal interaction occurs when the synthetic lethal partner is disrupted in the field of ncRNAs. ncRNAs are RNAs that do not encode proteins but have roles in a variety of processes such as modulating DNA transcription, regulating mRNA degradation, and they act as miRNA sponges and interact with DNA, RNA, or proteins [23]. Modulating ncRNAs can further affect their regulation of cellular responses and signaling pathways, which makes them potential targets for drug development.

In this study, we discussed therapeutic strategies targeting p53, ranging from direct targeting to synthetic lethal interactions with mutant p53. Additionally, as research into the functions of ncRNAs continues to progress, we addressed the synthetic lethal targets of mutp53 in the field of ncRNAs.

## Targeting p53 for cancer therapy

Numerous studies elucidated the roles of p53 in tumor progression since its discovery forty years ago. However, mutant forms of the tumor-suppressor p53 not only lose their tumor-suppressive properties but also frequently acquire tumor-promoting properties [2]. The development of p53-targeted drugs is particularly difficult because the agent must specifically target mutp53 in cancer cells while having no effect on normal cells harboring wtp53 [24]. Additionally, multiple p53 mutations result in various mutp53 protein structures that are difficult to target [25]. Major therapeutic strategies targeting p53 can be classified into two categories based on their p53 status: restoring wtp53 functions and eradicating mutp53 [8, 26–28].

### Reactivating suppressed p53 functions

While intact *TP53* is present in some cancers, the tumor suppressor is always inhibited via a variety of mechanisms. MDM2 is the major negative regulator of p53, which prevents p53 from entering the nucleus, inhibits its DNA binding, and promotes p53 proteasomal degradation [29, 30]. Genetic amplification is the most frequent genomic alteration of MDM2, which was first found in soft-tissue sarcoma [31]. It was discovered that amplification and overexpression of MDM2 were mutually exclusive with p53 mutation [32]. Oliner et al. discovered that MDM2 overexpression involved intact p53 across cancer types in a study using The Cancer Genome Atlas (TCGA) database [33]. Thus, inhibiting MDMs in cancers with wtp53 is an intriguing therapeutic strategy that has been successfully applied in clinical settings (Table 1). Since the discovery of a class of cis-imidazoline analogues known as nutlins that inhibit p53-MDM2 binding, MDM2 inhibitors have been extensively studied as a targeted treatment for patients with wtp53 [12, 34]. Nutlin-3a, a preclinical drug, inhibits tumor growth by reactivating wild-type p53, whether used alone or in combination with other therapies [35–37]. Due to the promising results of in vitro studies, clinical trials were conducted to assess the efficacy and safety of the derivative of nutlins, RG7112 (RO5045337) [38]. The majority of patients who accepted treatment with RG7112 had a stable disease. While nutlins can strongly activate wtp53 in tumors overexpressing MDM2, they are unable to activate the p53 pathway in cancers overexpressing MDMX due to subtle differences in the N-terminal p53-binding pocket of MDMX [39]. ALRN-6924 was the only dual MDM2 and MDMX inhibitor to reach clinical trials after preclinical investigations revealed a considerable antitumor effect [40, 41]. Since MDM2 and MDMX have distinct anti-p53 activities, dual antagonists targeting both p53-MDM2 and p53-MDMX may have a greater effect than inhibiting either pathway alone.

**Table 1:** Clinical trial agents targeting p53 negative regulators

ClinicalTrial.gov identifier and reference	Study design	Intervention and sample size (*n*)	Outcomes (*n*)	Toxicities (*n*)	Conclusions
NCT02016729; Erba et al. [146]	Non-randomized phase 1, open label	AMG232 (26) AMG232 + trametinib (10)	MLFS (4) CR (1), PR (1)	AEs (25) AEs (10)	AMG232 is activate and tolerable
NCT01164033, Patnaik et al. [147]	Randomized phase 1, open label	RG7112 single dose (23) RG7112 multiple dose (53)	N/A SD (6)	AEs (16), SAEs (2) AEs (52), SAEs (13)	High-dose consecutive is superior
NCT00623870; Andreeff et al. [61]	Phase 1, open label	RG7112 for AML (96) RG7112 for CML (20)	CR (2), CR_p_ (1), PR (1), MLFS (1), MTD (1500 mg BID), DLTs (3) PR (1), SD (15), MTD (N/A), DLTs (N/A)	AE grade ≥ 3 (86), AE grade ≥ 4 (34)	RG7112 is activated but need combination therapy
NCT02098967; Uy and Razak et al. [148, 149]	Non-randomized phase 1, open label	RG7775 for AML (26) RG775 for solid tumor (41)	CR (2), PR (2), HI/SD (7), MTD (200 mg), DLTs (4) SD (17), MTD (110 mg), DLTs (5)	AEs (25), SAEs (22) AEs (40), SAEs (6), death (2)	No improvement of safety
NCT02264613; Saleh et al. [150]	Non-randomized phase 1/2, open label	ALRN-6924: 0.16–4.4 mg/kg (41) ALRN-6924: 0.32–2.7 mg/kg (41)	CR (1), PR (1), SD (12), DLTs (5) CR (1), PR (8), SD (8), DLTs (0)	SAEs (7) SAEs (1)	ALRN-6924 is activated and tolerable
NCT01462175; Italiano et al. [151]	Non-randomized phase 1, open label	RG7388 QW*3 (36) RG7388 QD*3 (15) RG7388 QD*5 (34)	SD (10), MTD (1600 mg BID) SD (5), MTD (500 mg BID) SD (8), MTD (500 mg QD)	AEs (10), death (2) AEs (12) AEs (27), death (4)	RG7388 induces durable SD
NCT01773408; Yee et al. [44]	Non-randomized phase 1, open label	Idasanutlin (46) Idasanutlin-C (76)	CR_c_ (7) CR_c_ (21)	AE grade ≥ 3 (31), SAEs (24), death (11) AE grade ≥ 3 (69), SAEs (48), death (16)	Idasanutlin is activated and safety
NCT02545283; Papai et al. [152]	Non-randomized phase 1, open label	[^13/14^C]-label idasanutlin (8)	SD (1)	N/A	Hepatic impair alters idasanutlin exposure
NCT01760525; Bauer et al. [153]	Non-randomized phase 1, open label	CGM097 400 mg QW*3 (31) CMG097 300–700 mg QW*3 (20)	PR (1), SD (10) SD (9)	AE grade ≥ 3 (25), AE grade ≥ 4 (12)	CMG097 is tolerable with partial efficacy
NCT02143635; Jeay et al. [154]	Non-randomized phase 1, open label	HDM201 daily (20) HDM201 QW*3 (24)	N/A	N/A	Intermittent regimen is better

MLFS: morphologic leukemia-free state; CR: complete response; PR: partial response; AEs: adverse events; SD: stable disease; CR_p_: complete remission without platelet recovery; MTD: maximum tolerated dose; BID: twice daily; DLTs: dose-limiting toxicities; HI: hematologic improvement; SAEs: serious adverse events; QW: once weekly; QD: once daily; CR_c_: composite complete remission

While MDM2 inhibitors demonstrate a modest clinical response, the adverse events associated with their on-target effects should be considered [42]. Thus, combining MDM2 inhibitors with therapies such as chemotherapy, BCL2 inhibitors, CDK inhibitors, immunotherapy, or PI3K, MEK, and FLT3-ITD pathway inhibition is a better approach to reduce adverse events and improve therapeutic efficacy [43]. A majority of studies were conducted to determine the safety, pharmacokinetics, pharmacodynamics, and antitumor efficacy of these drugs. According to the results of a phase I study, when RG7388 (idasanutlin, RO5503781) was combined with cytarabine, the complete remission rate in patients with TP53wt AML was higher (35.6%) than when it was given alone (NCT01773408) [44].

### Making mutant p53 functional again

Since p53 is preferentially mutated in cancers, the treatment preventing p53 from degradation only works in cancer harboring wtp53, which limits its clinical application. Directly targeting mutp53 may have more application possibilities. However, restoring mutp53 therapeutically is more difficult than disrupting the p53-MDM2 interaction. Theoretically, the restoration of mutp53 is as follows: (1) mutp53 exhibits wild-type activity at permissive temperatures, (2) a second-site suppressor mutation can adapt to deleterious mutations and restore wild-type activities, and (3) a synthetic peptide, such as CDB3, derived from the p53-binding loop of 53BP2, can bind to the p53 core domain and rescue the DNA-binding ability of mutp53 [45–47]. The discovery of the first p53 reactivator, CP-31398, bodes well for the development of a mutp53 reactivator (Table 2). Several of these compounds have entered clinical trials, including APR-246 (eprenetapopt) in combination with azacytidine for myelodysplastic syndromes (MDS) with mutp53, which demonstrated a significantly higher rate of complete remission (CR) in patients with only TP53 mutation (NCT 03072043) [48].

**Table 2:** Compounds with the ability to reactivate mutp53

Compound name	Mechanism	Mutant p53 target	References
CP-31398	CP-31398 binds to the denatured DNA-binding domain of mutp53 and restores nature p53 conformation	V173A, S241F, R249S, R273H	[155]
PRIMA-1	PRIMA-1 enhances wtp53 stability at 37 °C, induces a conformational change of mutp53 and restores their DNA binding ability	R175H, R273H	[156]
APR-246	APR-246 enhances wtp53 stability at 37 °C, induces a conformational change of mutp53 and restores their DNA binding ability	R175H, R273H	[156]
MIRA-1	MIRA-1 prevents unfolding of wtp53 and mutp53 and restores native wtp53 conformation	R175H, R248Q, R273H	[157]
STIMA-1	STIMA-1 binds to the core domain of mutant p53 and results in the stabilization of wtp53 conformation	R175H, R273H	[158]
PK11000	PK11000 increases the Tm of mutp53 to promote correct fold	Y220C	[159]
ZMC1	ZMC1 provides addition Zn^2+^ to cancer with mutp53 for proper folding	R175H, R172H	[160]
COTI-2	COTI-2 converts mutp53 to wild-type form	R175H	[161]
pCAPs	pCAPs binds to the core domain of mutp53	I195T	[47]
Reacp53	Reacp53 prevents mutp53 amyloid aggregation	P53 252–258 amyloid zipper structure	[162]
RETRA	RETRA releases p73 from mutp53-p73 complex	P73	[163]
PK083	PK083 binds and stabilizes p53-Y220C to restore its transcriptional activity	Y220C	[164]
P53R3	P53R3 restores sequence-specific DNA-binding ability of several mutp53	R175H, M237I, R273H	[165]
SCH529074	SCH529074 enables mutp53 to bind to a consensus p53 DNA-binding site	R175H, L194F, R248W, R249S, R273H	[166]
PK7088	PK7088 selectively binds to the specific surface cavity of p53 Y220C to destabilize it and restores wtp53 conformation	Y220C	[167]
Stictic acid	Stictic acid blocks the pocket between loop L1 and sheet S3 of p53 core domain and reactivates mutp53	R175H, G245S	[168]
Chetomin	Chetomin promotes Hsp40 expression and the p53-Hsp40 binding to restore wtp53 conformation	R175H	[169]
RITA	RITA restores transcriptional activity of mutp53	I254D, R175H, R213Q, Y234H, R248W/Q, R273H, R280K	[170]
WR1065	WR1065 restores temperature-sensitive mutp53 native conformation	V272M	[171]
Adamantyl isothiocyanates	Adamantyl isothiocyanates rescues mutp53 R206K and R273H and results in the upregulation of canonical wtp53 targets and ATM phosphorylation	R280K, R273H	[172]

p53 reactivators are classified in a variety of ways due to the multiple chemical classes or their overlapping roles [14, 49]. The primary activities of p53 rescuers include: (1) stabilization of the wtp53 structure, (2) refolding or preventing misfolding of mutp53, (3) restoration of the DNA-binding ability of mutp53, and (4) promoting the expression of full-length protein from mRNAs with nonsense mutations. Although multiple p53 reactivators have been developed, only two drugs have entered clinical trials, APR-246 and COTI-2. One of the challenges in drug development stems from the p53-independent toxicities of these compounds, which may also contribute to their antitumor activity. For instance, APR-246 induces oxidative stress by converting thioredoxin reductase 1 to a NAPDH oxidase [50]. Thus, more targeted p53 reactivators are needed to minimize toxicity and improve the therapeutic window.

### Depletion of mutant p53

In addition to reactivating mutp53, selective targeting of mutp53 proteins may also exhibit an antitumor effect [15]. This compound development strategy is based on the following observations: (1) depletion of mutp53 by siRNA or shRNA can suppress the mutp53-mediated malignant progression and (2) mutp53 is inherently unstable [51–53]. Therefore, the cornerstone of this strategy is to restrict the expression of mutp53 and promote the degradation of mutp53 (Table 3).

**Table 3:** Compounds directly target mutp53

Compound name	Mechanism	Targets	References
Hsp90 inhibitor	Hsp90 inhibitor prevents Hsp90 chaperone from binding to mutp53 and promotes mutp53 proteins degradation	R175H, L194F, R248Q, R273H, R280K, R172H (mouse)	[173]
HDAC inhibitor	HDAC inhibitor inhibits HDAC-regulated transcription and disrupts HDAC6/Hsp90/mutp53 complex	R175H, R280K, V247F/P223L	[56]
Statins	Statins inhibits the interaction between mutp53 and DNAJA1 to induce CHIP-dependent p53 degradation	V157F, R172H, R175H, Y220C, R248W, R273H, R280K	[174]
Gambogic acid	Gambogic acid increases wtp53 proteins levels, inhibits mutp53-Hsp90 complex and induces CHIP-mediated degradation	R175H, G266E, R273H, R280K	[58]
Spautin-1	Spautin-1 inhibits macroautophagy to induce mutp53 degradation via chaperone-mediated autophagy	P98S, P151H, A161T, R175C, R175D, R175H, L194F, S227K, S227R, G245C, R248L, R248W, E258K, R273H, R273L, R280K, and R282W	[99]
NSC59984	NSC59984 promotes MDM-mediated mutp53 protein degradation and stimulating p73	R175L, R175H, S241F, R273H/P309F	[175]
Disulfiram (DSF)	Disulfiram catalyzes both wtp53 and mutp53 through the 26S proteasome pathway	R273H	[176]

Histone deacetylase inhibitors (HDACis) are a type of antitumor agent that inhibits histone deacetylases (HDACs) and thereby regulate gene expression [54]. The expression level of mutp53 can be transcriptionally reduced by HDACis [55]. Additionally, HDACis also disrupt the HDAC6/Hsp90/mutp53 chaperone complex to destabilize mutp53 proteins and promote their degradation [56]. Targeting another mutp53 protein stabilizer, Hsp90, via an Hsp90 inhibitor, can induce apoptosis in cancers with p53 deficiency [57]. Additional compounds were discovered to be capable of promoting mutp53 degradation. For example, gambogic acid, a traditional Chinese medicine, promotes mutp53 proteasomal degradation via the chaperone-associated ubiquitin ligase carboxy terminus of Hsp70-interacting protein (CHIP) [58]. However, these agents exhibit a pan-antitumor effect apart from degrading mutp53 [59, 60]. The act of depleting p53 within their antitumor effect should be further discussed, and the screening of agents selectively targeting mutp53 is needed.

### The challenges of p53-targeted therapy

**Fig. 2:**
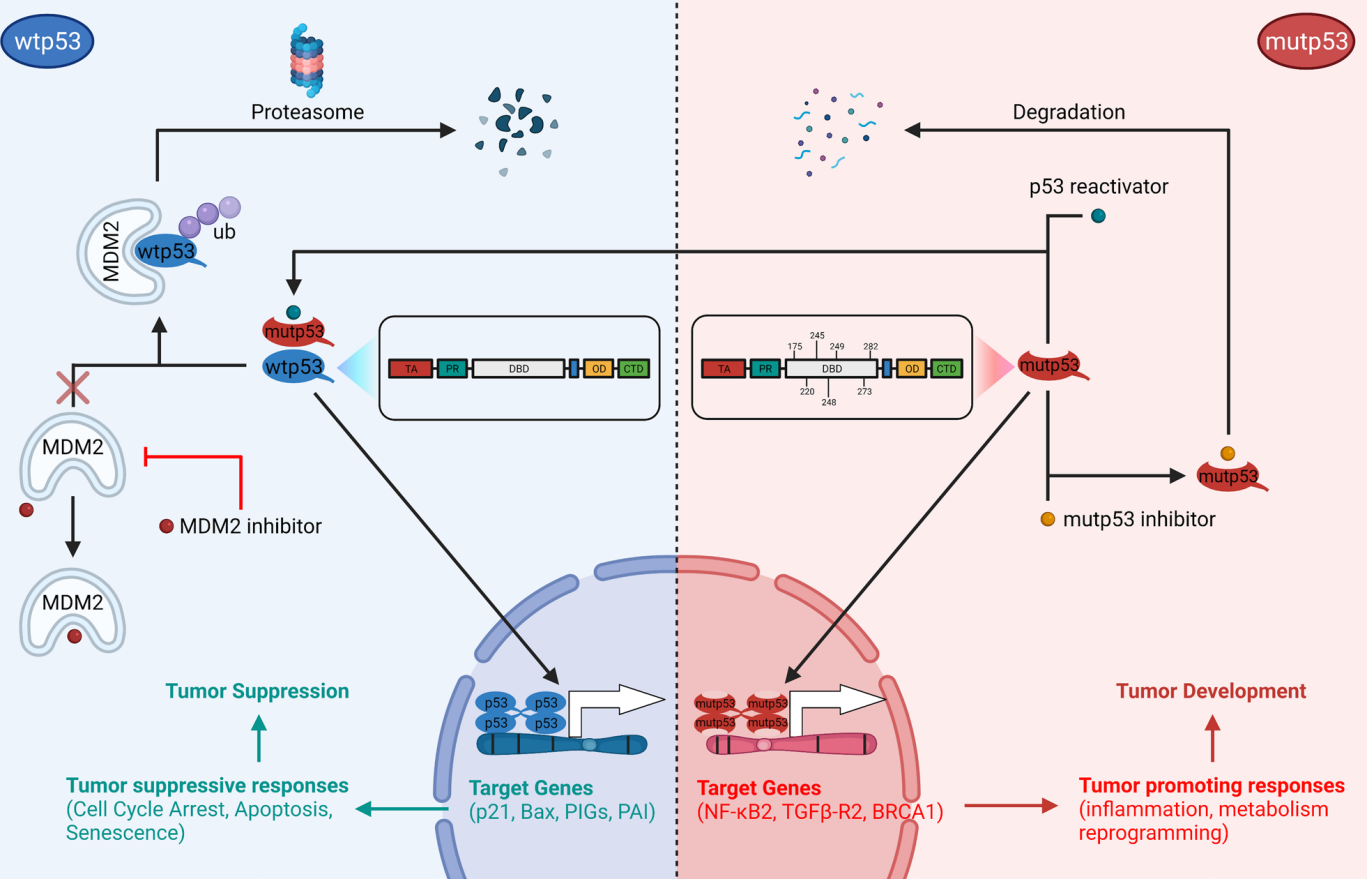
Multiple anticancer therapeutic strategies targeting p53. The p53 protein, encoded by the *TP53* gene, consists of five functional regions: TA, PR, DBD, OD, and CTD. (Left) After being imported into the nucleus and tetramerized, wtp53 proteins acquire the ability to bind with their target genes (e.g., p21, Bax, PIGs, PAI) to induce tumor-suppressive responses (cell cycle arrest, apoptosis, and senescence) to suppress tumor initiation or progression. The ubiquitin E3 ligase MDM2 directly binds to p53 proteins and promotes proteasomal degradation. For cancers containing wtp53, MDM2 inhibitors (e.g., AMG232 and RG7388) prevent p53 from proteasomal degradation and promote its tumor-suppressive functions via disrupting the p53-MDM2 protein–protein interaction. (Right) Most mutations of mutp53 proteins occur in the DBD regions, including several hotspot mutation sites (175, 220, 245, 248, 249, 273, 282). Mutp53 proteins lose the ability to bind with tumor-suppressive genes and even acquire functions to transcriptionally activate oncogenic genes (e.g., NF-κB2, TGFβ-R2, BRCA1) to induce tumor-promoting responses such as inflammation and metabolic reprogramming. Mutp53 reactivators target specific p53 mutations (e.g., APR-246 targets p53 R175H and R273H, and COTI-2 targets p53 R175H) to restore wtp53 functions. Additionally, mutp53 inhibitors directly bind to mutp53 (e.g., HDAC inhibitor for p53 R175H, R280K and V247F/P223L, and disulfiram for p53 R273H) to promote degradation. TA: transactivation domain; PR: proline-rich domain; DBD: DNA-binding domain; OD: oligomerization; CTD: carboxyl-terminal domain; Ub: ubiquitin

As mentioned previously, numerous strategies can be used to target wt/mutp53 in cancer therapy (Fig. 2). The MDM2/X inhibitor is effective for cancers with wtp53, which is reflected in clinical trials (Table 1). Additionally, we should recognize that directly targeting mutp53 is difficult due to the structural diversity of mutp53. It is exceedingly difficult to discover a compound that can target all mutp53. Moreover, because the p53 pathway is quite complex, and restoring p53 function in normal tissue can result in unpredictable adverse events. For instance, RG7112 is associated with at least one adverse event that is frequently associated with hematological toxicity in patients [61]. In general, strategies that directly target mutp53 require that the agents have a higher affinity for mutp53 in order to have a greater antitumor effect and fewer adverse events.

## Synthetic lethality with p53: a strategy that makes p53 druggable

**Fig. 3:**
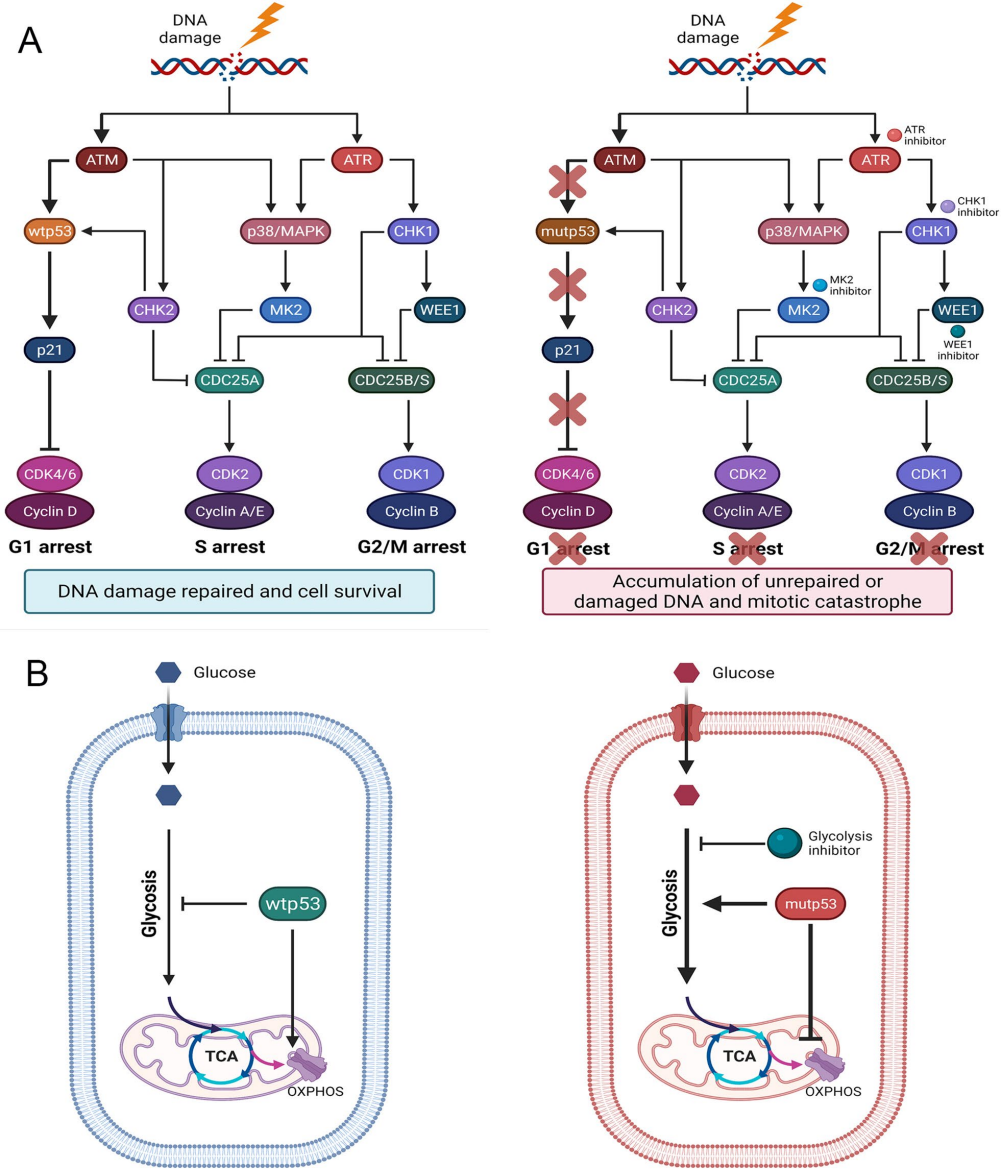
Synthetic lethality with mutp53 LOF and GOF. **A** Wtp53 maintains the survival-promoting pathways when cells undergo stress. Mutp53 loses these functions but activates compensatory pathways to protect cancer cells from lethal stresses. Thus, these compensatory pathways become vulnerable in cancers as these pathways are less dependent on normal cells. Taking the role of p53 in cell cycle arrest as an example, in response to DNA damage, wtp53 can activate p21 to induce G1 arrest to repair DNA damage (left). Under conditions of p53 mutation, cancer cells rely more on S and G2 arrest for DNA repair. Inhibition of regulators of S and G2 arrest results in the accumulation of unpaired DNA and mitotic catastrophe (right). **B** For mutp53 GOFs, the target genes upregulated by mutp53, which are usually silenced when p53 is not mutated, might be the crucial factors promoting tumor progression. Targeting these genes can selectively suppress the cancer progression of cancers with mutp53. Regarding energy metabolism, wtp53 inhibits glycolysis and promotes OXPHOS (left), and mutp53 acquires the opposite functions to promote glycolysis and inhibit OXPHOS (right). Herein, targeting the enhanced glycolysis induced by mutp53 can be developed for synthetic lethal approaches

In comparison with p53-targeted agents, the synthetic lethal method's efficacy is less dependent on the mutp53 structure, indicating that it could be used in a broad range of conditions. Different strategies may be used depending on the mutp53 LOFs and GOFs. As mentioned previously, targeting the compensatory induced G2 arrest is of great interest for mutp53 LOFs. Suppressing acquired oncogenic signaling allows for the selective elimination of p53 GOF mutations. To be precise, synthetic lethal pathways involving mutp53 are numerous and vary according to the functional alteration induced by mutp53 (Fig. 3).

### Targeting mutp53 with cell cycle arrest

Due to the increased reliance on intra-S and G2 arrest in cancers with mutp53, the regulators of the intra-S and G2 checkpoints (e.g., ATR, CHK1, MK2, and Wee1) have been identified [62–65]. ATR acts as a recognizer of specific single-stranded DNA sites and phosphorylates CHK1 to regulate the cell cycle and DNA damage response [66]. Reaper et al. first identified the synthetic lethal interaction between ATR and p53 using the selective ATR inhibitor VE-821 (Table 4) [67]. M6620 was the first ATR inhibitor to be tested in humans. The recent M6620 phase 2 clinical trial demonstrated a superior effect of M6620 and gemcitabine combination therapy (median progression-free survival, 22.9 weeks, 90% CI 17.9–72.0 weeks) compared to gemcitabine monotherapy (median progression-free survival, 14.7 weeks, 90% CI 9.7–36.7 weeks), indicating that ATR inhibitors can potentially enhance the effect of current chemotherapy [68, 69]. Additionally, prexasertib, a CHK1 inhibitor, demonstrated antitumor activity in several high-grade serous ovarian cancer patient-derived xenograft (PDX) models [70]. Moreover, prexasertib treatment may synergize with PARP inhibition and resensitize olaparib-resistant models to olaparib treatment. This result demonstrated that inhibiting G2 arrest in cancers harboring a p53 mutation has significant clinical implications. However, three clinical trials of CHK1 inhibitors (NCT01870596, NCT02797964, and NCT02797977) have been completed for ATR inhibitors compared to null. The reason may derive from the fact that ATR inhibition can result in significant defects in chromosomal segregation in normal cells, and the molecular weight of ATR is too large for compound screening [62, 71].

**Table 4:** Synthetic lethal partners with mutp53

Synthetic lethal partner	Mechanism	References
*ATR*	ATR inhibitors target ATR/CHK1 pathway to suppress G2 arrest in p53-deficient cancers	[68, 69, 177]
*CHK1*	CHK1 inhibitors target CHK1 to suppress G2 arrest in p53-deficient cancers	[70]
*p38MAPK/MK2*	MK2 regulates of G2/M and S-phase checkpoint in p53-mutated/p53-deficient cancers in response to DNA damage	[64, 72]
*WEE1*	WEE1 plays a crucial role in the G2/M transition for p53-mutated cancers	[76, 77]
*PLK1*	PLK1 is required for mitosis entry by activating CDC25 and inhibiting both WEE1 and PKMYT1	[73, 79]
*PIP4K2B*	High level of PI5P4Ks promotes glucose uptake to support glucose metabolism and enhances NADPH generation to mediated ROS response in the condition of p53 mutation/deletion	[89]
*HK2*	HK2 regulates the transform from phosphorylating glucose into glucose-6-phosphate to elevate glycolysis for glycolysis	[88, 178]
*mTOR*	mTOR-induced inhibition of autophagy prevents mutp53 proteins from autophagy-mediated degradation	[96]
*PDGFRβ*	PDGFRβ maintains the premetastatic phenotype for pancreatic ductal adenocarcinoma with p53 R172H	[103]
*PLA2G16*	Mutp53 binds to the promoter of *PLA2G16* to promote metastatic phenotype	[104]

Other G2 arrest regulators may also act as mutp53 synthetic lethal partners. The p38MAPK/MK2 pathway, which regulates the G2/M checkpoint, is activated in response to DNA damaging agents [72]. A recent study demonstrated the induction of synthetic lethality using a new cytarabine analogue, F-Se-Ara-C, that targets MK2 in prostate cancer with p53 mutation [64]. Cdc25B-mediated G2 arrest was disrupted by MK2 depletion only in p53-deficient cells, but not in wtp53-carrying cells, confirming the synthetic lethality interaction between p53 and MK2 [72]. Polo-like kinase 1 (*PLK1*) and *WEE1* are two other cell cycle regulators that have synthetic lethal interactions with p53 [65, 73]. The WEE1 kinase family is composed of three kinases: WEE1, WEE1B, and membrane-associated tyrosine- and threonine-specific cdc2-inhibitory kinase (PKMYT1) [74]. Among them, WEE1 and PKMYT1 are critical G2/M transition regulators, as the former inhibits mitotic entry by phosphorylating CDK1 on Tyr15, whereas the latter promotes mitotic entry via dual targets on Tyr15 and Thr14. [75]. MK-1775, a WEE1 inhibitor, has been discovered to have a great ability to radiosensitize human cancer cells, which only occurs in p53-deficient tumors [76]. The clinical trial on head and neck squamous cell cancer (HNSCC) using MK-1775, cisplatin, and docetaxel (NCT02508246) combined treatment validated the effectiveness and safety of this synthetic lethality between *WEE1* and *TP53* [77]. PLK1 is required for mitotic entry by activating CDC25 and inhibiting both WEE1 and PKMYT1 [78]. Inhibition of PLK1 by BI2536 significantly suppresses cells with p53 mutation [73]. However, the PLK1 inhibitor BI2536 had a low response rate and induced severe adverse effects in patients resulting in poor efficacy [79]. The reason might be associated with the multiple roles of PLK1 in addition to regulating the G2/M checkpoint, including centrosome coordination, regulating chromosome segregation, and facilitating DNA replication [78]. The distinct outcomes of targeting regulators during G2 arrest suggest that a greater understanding of mutp53 and the G2 arrest is required and that more selective regulators are preferable candidates.

### Targeting mutp53 with energy metabolism

The involvement of p53 in glycolysis and oxidative phosphorylation (OXPHOS) is well recognized [80]. p53 suppresses glycolysis by inhibiting multiple regulators in the glycolytic pathway. For example, p53 can limit glucose uptake by inhibiting the expression of glucose transporter 1/4 (*GLUT1/4*) directly or *GLUT3* indirectly via the IKKβ/NF-κB/GLUT3 pathway [81, 82]. p53 stimulates OXPHOS via multiple pathways, including transactivating cytochrome c oxidase 2 (*SCO2*) to maintain cytochrome c oxidase assembly and upregulating apoptosis-inducing factor (*AIF*) to maintain mitochondrial complex I integrity [83, 84]. Reprogramming of energy metabolism from OXPHOS to glycolysis even under aerobic conditions (Warburg metabolism) is one of the hallmarks of cancer [85]. The majority of mutant p53 loses its ability to maintain metabolic homeostasis and even acquires additional functions to promote Warburg metabolism. Based on this phenomenon, combining synthetic lethality with energy metabolism is extremely appealing.

Glucose uptake is the first rate-limiting step of glycolysis. Compared to the suppressive effect of p53 on GLUT, mutp53 promotes GLUT expression by augmenting glucose uptake in cancer cells [86]. For instance, Zhang and colleagues discovered that lung cancer cells expressing R175H, R248Q, and R273H mutant p53 exhibited an enhanced Warburg effect via activation of the RhoA/ROCK pathway, thereby increasing GLUT1 expression and membrane localization [86]. Inhibition of GLUT and RhoA suppressed tumor progression, but targeting RhoA was more tumor-specific as it only impacted mutp53-mediated glycolysis, which had less effect on normal cells. Hexokinase-II (HK2), the first rate-limiting enzyme of glycolysis, was transcriptionally downregulated by wtp53, and its mRNA level was also reduced by wtp53 [87, 88]. p53 deficiency enables the upregulation of HK2, and HK2 knockdown significantly reduces cancer cell proliferation [88]. However, HK2 also maintains glycolysis in normal cells, which leads to the perplexity of inhibiting HK2 for cancer therapy. Additionally, Emerling et al. found a synthetic lethal relationship between type 2 PIP kinase genes (*PIP4K2B*) and p53 mutation/deletion [89]. Under the context of p53 loss, phosphatidylinositol-5-phosphate 4-kinase β (PI5P4Kβ, encoded by *PIP4K2B*) plays a crucial role in maintaining glucose metabolism and reactive oxygen species (ROS) homeostasis. The functions of PI5P4Kβ were unnecessary in a cell line with wtp53. In addition to the factors regulating glycolysis, mutp53 also regulates glycolysis indirectly by ncRNAs. For example, lncRNA AGPG is negatively transcriptionally regulated by p53, which regulates glycolysis by binding to and stabilizing phosphofructo-2-kinase/fructose-2,6-biphosphatase 3 (PFKFB3), a rate-limiting enzyme in glycolysis [90]. However, mutp53 loses this transcription suppressive function, leading to the upregulation of AGPG and promotes glycolysis for cancer progression. This phenomenon indicated that targeting ncRNAs may also induce synthetic lethality in p53-mutated cancers. Taken together, both altered energy metabolism induced by oncogenic mutp53 GOFs and compensatory activated signaling for p53 LOFs were viable synthetic lethal strategies in the presence of p53 mutation.

Although mutp53 preferentially exhibits glycolysis-promoting functions, several cancer cells with mutp53 display enhanced mitochondrial functions. Eriksson et al. found that H1299 cells induced with p53 R175H and R273H showed enhanced mitochondrial respiration capacity [91]. This finding suggested the possibility of targeting OXPHOS for cancer therapy with mutp53. Additionally, inhibiting OXPHOS could be regarded as a combination regimen with glycolysis inhibitors. This approach showed feasibility, as cotreatment with 2-deoxyglucose (glycolytic inhibitor) and metformin (OXPHOS inhibitor), had a significant effect on prostate cancer cells compared to monotherapy [92]. However, in our opinion, targeting OXPHOS might be a riskier strategy as OXPHOS is the major energy resource of normal cells. Moreover, Warburg metabolism is a common metabolic alteration in cancer cells instead of an advantage of cancers with p53 mutations. Herein, screening synthetic lethal partners with mutp53 in the field of energy metabolism should integrate the specific genetic alteration of mutp53.

### Other potential synthetic lethal pathways with mutp53

#### Targeting mutp53 with autophagy

Autophagy is a housekeeping process that controls protein and organelle quality and recycles intracellular components such as misfolded proteins and dysfunctional mitochondria as alternative resources to maintain normal biological activities, particularly under conditions of nutritional deprivation [93]. In tumors, autophagy has a dual effect: it inhibits tumor formation while also promoting tumor growth once it has begun [94]. Autophagy is thought to be suppressed by mutp53. Several recent studies have identified that mutp53 R175H, R273H, and R273L can inhibit autophagy by blocking AMPK, activating mTOR, and stabilizing HIF-1 proteins [95]. Additionally, mutp53 represses the NF-κB-mediated expression of atg12 to impact autophagy by interacting with the p50 NF-κB subunit [96].

Autophagy inhibition can be viewed as a p53 GOF that promotes tumor progression by reducing mutp53 degradation via the autophagy pathway [97]. The canonical AMPK/mTOR pathway is regarded as the core signaling cascade of mutp53-mediated suppression of autophagy [98]. Pharmacological induction of autophagy by mTOR inhibition or AMPK activation has been shown to have potential therapeutic value, and the activation of autophagy induced by mutp53 makes cancer cells more sensitive to the mTOR inhibitor everolimus, especially in cancers with p53 mutations [96]. Inhibiting mTOR to induce autophagy may be a potential synthetic lethal approach to mutp53 as mTOR is usually activated in cancer cells. Several strategies to deplete mutp53 also rely on autophagy; for example, spautin-1 promotes chaperone-mediated autophagy to degrade mutp53 [99]. As autophagy initiation is quite complicated, we can hypothesize that drugs that affect the autophagy process rather than its constituents are synthetically lethal for cancers with p53 mutations.

#### Targeting mutp53 activated invasion and metastasis

Although exploiting the strategies to selectively kill tumor cells with mutp53 is appealing, targeting mutp53-activated invasion and metastasis to suppress tumor progression is also attractive. Mutp53 has been demonstrated to have metastatic potential by promoting epithelial–mesenchymal transition (EMT), altering the extracellular matrix (ECM), and inhibiting its binding partner p63/p73 to indirectly modulate the genes that control metastasis and invasion [100–102]. Mutp53 can facilitate invasion by increasing receptor expression and activating downstream signaling pathways, similar to how TP53 R175H and R273H promote metastasis via EGFR recycling. It is plausible that metastatic cancers in their advanced stages rely on mutp53-mediated invasion and metastasis.

A murine model study revealed that sustaining the premetastatic phenotype of pancreatic ductal adenocarcinoma (PADC) requires sustained expression of p53 R172H, which increases platelet-derived growth factor beta (PDGFR), and inhibition of PDGFR with imatinib significantly prevents PDAC metastasis in vivo [103]. Another study in a murine model found that *PLA2G16*, a phospholipase that can catalyze phosphatidic acid into metastasis-promoting lysophosphatidic acid and free fatty acid, was upregulated in p53 R172H mutant osteosarcomas by binding to its promoter at E26 transformation-specific binding motifs [104]. Knockdown of *PLA2G16* or ETS specifically weakens the metastatic potential of cancer [104].

## Noncoding RNAs: expanding the potential synthetic lethality network of p53

Genes encoding proteins account for only 1–2% of the human genome, leaving numerous ncRNAs to be exploited for cancer therapy [23]. ncRNAs can be classified into two types based on size: small RNAs such as microRNAs (miRNAs) and long noncoding RNAs (lncRNAs) at least 200 nucleotides in length [105]. Over the last few decades, researchers have discovered that ncRNAs participate in various cellular and molecular mechanisms, such as the regulation of gene expression, chromatin modification, and lncRNA-protein interactions [23, 106, 107]. p53 also plays an important role in the regulation of ncRNAs including miRNAs and lncRNAs [108–110]. Mutation of p53 could lead to a dysregulated wtp53/ncRNA network and active mutp53-mediated ncRNA alteration. Therefore, the development of a synthetic lethal approach with mutp53 is theoretically feasible in the field of ncRNAs.

### Mutp53 and microRNAs

The most prevalent and well-studied small ncRNA type are miRNAs, which have a length of approximately 20 nt [111]. The major functions of miRNAs are typically associated with inhibiting the translation or promoting the degradation of their target mRNAs [106]. Thus, altered miRNA levels can further influence protein concentrations via a posttranscriptional mechanism. Wtp53 can regulate miRNA levels by directly activating transcription or promoting maturation [112]. In contrast, p53 GOF mutations result in the dysregulation of the wtp53/miRNA axis and upregulation of the expression of other miRNAs to confer additional oncogenic abilities, such as metastasis and somatic cell reprogramming [113, 114]. Moreover, several studies have recently focused on circulating miRNAs to explore their diagnostic and prognostic potential, indicating the potential clinical application of miRNAs [115]. Therefore, targeting mutp53-specific miRNAs may become an effective approach for cancer therapy.

The miR-34 family is composed of three members miR-34a and miR-34b/c, which are the first reported miRNAs directly regulated by p53 [116]. The activation of p53 upregulates miR-34 expression, which is synergistic with the antitumor effect of p53. It has been well demonstrated that miR-34 expression levels are lower in cancer tissues than in normal tissues, such as intestinal, breast, and lung cancers [117–119]. Thus, miR-34a is an appropriate target for cancer therapy. For example, Park et al. found low expression of miR-34a in MCF7/ADR cells, and ectopic expression of miR-34a significantly suppresses tumor growth by inhibiting Notch1 [118]. MiRNAs as cancer therapeutic targets are now possible due to the development of miRNA delivery systems that utilize viral or nonviral vectors [120]. In phase I clinical trial using MRX34, the application of a miRNA-associated therapeutic drug was first explored (NCT01829971) [121]. However, due to the severity of immune-mediated adverse events, this study was halted. One reason for this may be due to MRX34's on-target effect in normal tissues, which results in an induced miR-34a-mediated response. Even though the study was unsuccessful, it established a proof of concept for miRNA-based therapy.

In addition to targeting miRNAs dysregulated by loss of wild-type p53 functions, targeting mutp53-mediated miRNAs is also attractive. As is the case with the miRNAs summarized in Table 5, miRNAs mediated by mutp53 have been linked to tumor progression. Luo et al. found that p53-targeted miR-223-3p expression was decreased in p53-mutated lung cancers because mutp53 could bind to the miR-223-3p promoter and inhibit its expression [122, 123]. However, miR-223-3p acted as a tumor suppressor via the miR-223-5p-mutp53 feedback loop, because mutp53 was a target of miR-223-3p [123]. Treatment with the miR-223-3p agomir significantly suppressed the progression of lung cancer in vivo. Numerous studies have established the tumor-suppressive properties of miR-223-3p, and based on this study, we are convinced that miR-223-3p is a component of synthetic lethality to mutp53.

**Table 5:** Noncoding RNAs regulated by mutp53

Target	Mechanism	Cancer type	References
miR-130b	miR-130b promotes EMT by ZEB1	Endometrial cancer	[179]
miR-30d	miR-30d impacts on the sector to promote ECM for metastasis	Breast cancer	[124]
miR-21-3p/miR-769-3p	p53 R273H activates fibroblasts by exosomal miR-21-3p	Lung cancer	[113]
miR-1246	Cancers with mutp53 shed exosomes containing miR-1246 induce a miR-1246-dependent reprogram of TAMs to a tumor-suppurative status	Colon cancer	[180]
miR-34	This tumor-suppressive miRNA is usually downregulated in p53-mutated cancers	Multiple cancers	[117, 181]
miR-223-3p	Mutp53 directly binds to the promoter region of miR-223-3p that can suppress tumor proliferation and migration	Lung cancer	[123]
miR-200	miR-200 suppressed by mutp53 losses the ability to inhibit metastasis	NSCLC	[182]
Let-7i	let-7i inhibits invasion and metastasis by repressing E2F5, LIN28B, MYC, and NRAS	Breast cancer	[52]
miR-142-3p	Upregulation of Dnmt1 promotes miR-142-3p hypermethylation of its locus	Pancreatic ductal adenocarcinoma	[183]
LincRNA-p21	LincRNA-p21 cannot be activated by mutp53 in response to DNA damage	HNSCC	[137]
AGPG	p53 mutations lose the transcriptionally suppress function to inhibit AGPG expression, and upregulated AGPG stabilizes PFKFB3 to promote glycolysis	Esophageal squamous cell carcinoma	[90]
MALAT1	MALAT1 acts as a bridge between mutp53 and ID4 to modulate *VEGFA* isoforms expression	Breast cancer	[138]
Lnc273-31/34	p53 R273H specific upregulates lnc273-31 to maintain self-renew of colorectal CSCs	Colorectal cancer	[184]
lncMIR205HG	Mutp53 recruited by NF-YA and E2F1 to the promoter of MIR205HG to upregulate lncMIR205HG to promote cancer progression	HNSCC	[185]
LINC00460	Upregulation of LINC00460 by mutp53 increases mutp53 levels through competitively binding to miRNAs targeting mutp53	Colorectal cancer	[186]
CircPVT1	CircPVT1 is upregulated by mutp53 in HNSCC patients to promote cancer proliferation by miR-497-5p	HNSCC	[187]
Circ-CCNB1	Circ-CCNB1 is suppressed in p53-mutated cancer, and expression of circ-CCNB1 activates the H2AX-dependent tumor suppressor Bclaf1 only in cancer cells harboring mutp53	Breast cancer	[140]

Mutp53 can also upregulate multiple oncogenic miRNAs to promote tumor progression. For example, mutp53 can induce the expression of miR-30d, which causes tubule vesiculation of the Golgi apparatus to enhance vesicular trafficking and secretion to promote metastasis [124]. Additionally, mutp53 boosts the release of miR-1246 into tumor stroma to support the formation of tumor-associated macrophages [125]. Targeting these mutp53-associated miRNAs can suppress tumor growth.

### Mutp53 with long noncoding RNAs and circRNAs

Compared to miRNAs, the characterizations and functions of lncRNAs, which are cell-type specific, are more complex. Although over 60,000 distinct lncRNAs have been predicted based on initiatives and databases such as ENCODE, GENCODE, LNCipedia, and lncRNome, the majority of these lncRNAs still need to be studied and annotated [126–129]. LncRNAs are categorized into four types according to their functions: signals, decoys, guides, and scaffolds [130, 131]. The structural flexibility of lncRNAs allows them to interact with DNA, RNA, or proteins via base pairing [130]. As an example of transcriptional regulation, a sequence-specific mature lncRNA transcript can have a direct effect on the expression of its adjacent genes, as demonstrated by the positive and negative regulation of X-chromosome inactivation by lncRNA Tsix and its antisense counterpart Xist. On the other hand, lncRNAs indirectly regulate transcription by decoying or guiding regulatory proteins. For example, the long noncoding RNA Gas5 acts as a decoy for DNA receptors, preventing metabolic genes from being transcribed. Additionally, lncRNA ANRIL directs polycomb repressive complexes 1 and 2 (PRC1 and 2) to regulate cell cycle-related gene expression. A scaffold function has been demonstrated by the finding that GClnc1 binds WDR5 and KAT2A histone acetyltransferases, thereby specifying the histone modification of mitochondrial superoxide dismutase 2 [132–135].

As a well-studied transcription factor, p53 also regulates the expression of lncRNAs to compose the p53 signaling network. It is reasonable that tumor-associated lncRNAs induced by p53 mutation can be exploited for cancer therapy. LincRNA-p21, which acts as a repressor of the p53 pathway and cannot normally be induced by mutp53 in response to DNA damage, was the first identified lncRNA transcriptionally induced by p53 [136]. A recent study conducted by Jin et al. showed that lincRNA-p21 can also be regulated by mutant p53 in cooperation with nuclear transcription Factor Y subunit α (NF-YA) to block JAK/STAT3 signaling to suppress tumor progression [137]. It is reasonable to be concerned that increasing the interaction between mutp53 and NF-YA may inhibit tumor progression in mutp53-positive cells via upregulation of lincRNA-p21. Apart from directly regulating the expression of lncRNAs, mutp53 can also modulate the expression of tumor promoter factors by cooperating with lncRNAs. MALAT1 is a lncRNA that regulates serine-/arginine-rich (SR) proteins in the nucleus and interacts with mutp53, IDH4, and SRSF1 to form a complex to promote VEGFA expression in breast cancer [138]. This complex does not exist in the presence of wtp53. Targeting the mutp53/IDH4/SRSF1/MALAT1 complex may provide a new therapeutic approach for mutp53 breast cancers.

CircRNA is a type of noncoding RNA that connects its 3’ and 5’ ends via “backsplicing” to form a single-stranded loop structure that is resistant to exonuclease-mediated degradation [139]. CircRNAs function as miRNA or RNA-binding protein (RBP) sponges to regulate gene expression or as scaffolds to aid protein complex assembly, interact with proteins to enhance their functions, and they can even be translated into peptides themselves [139]. Circ-Ccnb1 is downregulated in breast cancer and has tumor-inhibitory properties [140]. Surprisingly, ectopic expression of circ-Ccnb1 reduced proliferation only in cancers harboring mutp53. The interaction between circ-Ccnb1, wt/mutp53, H2AX, and Bclaf1 is thought to be the mechanism underlying this phenomenon. H2AX acts as a bridge for circ-CCnb1 to bind wtp53 or Bclaf1, but H2AX binds wtp53 more strongly. Increased circ-Ccnb1 binding increases wtp53 binding in p53 wild-type cells, thereby inhibiting the H2AX-dependent tumor-suppressor Bclaf1 from inducing apoptosis. Mutp53, on the other hand, is unable to bind to H2AX, resulting in Bclaf1-H2AX binding and apoptosis-inducing the proliferation of cancer cells. This interaction demonstrates the possibility of targeting circ-Ccnb1 to selectively kill cancer cells using mutp53 while sparing normal tissues.

Several approaches can be adopted to modulate lncRNAs: (1) employing promoter-targeted duplex RNAs to increase the expression of lncRNAs whose transcripts overlap gene promoters; (2) inhibiting lncRNA levels using antisense oligonucleotide (ASO) or siRNA-mediated knockdown; and (3) preventing the binding of lncRNAs to DNA, RNA, or proteins by utilizing small molecules/peptides [141]. However, no lncRNA drugs have entered clinical trials. The difficulties stem from the poor conservation of lncRNAs and the transferability of lncRNA studies from mouse models to humans.

## Future perspectives of mutp53-targeted cancer therapy

Due to the high frequency of p53 mutations, it is an appealing target for therapeutic strategies. The most convenient strategy is to directly target p53. In cancers where p53 is intact, inhibiting its negative regulator MDM2/X can activate the native tumor-suppressive function of p53. However, a significant limitation of this approach is the adverse event caused by widespread wtp53 activation in normal tissues [61]. It is preferable to develop a drug delivery system capable of transporting drugs specifically into tumors. For instance, nanomedicine-based therapy can significantly enhance drug efficacy while minimizing adverse effects [142]. Moreover, MDM2 inhibitor monotherapy is insufficient to completely suppress tumor progression. Thus, exploring a combination regimen of MDM2 inhibitors with other treatments may be a prospect for anticancer strategies [143]. For the strategies to reactive or deplete mutations, challenges arise from the determination of mutp53 protein structure [144]. Multiple p53 mutations necessitate the use of multiple agents directed against mutp53, let alone the specific and effective functions of the mutp53 reactivator/inhibitor.

In comparison with directly targeting p53, a synthetic lethal approach may be more widely used. As previously stated, p53 mutations are prevalent in a variety of cancer types [6]. LOFs derived from mutp53 activate alternative pathways that are compensatory for wtp53's survival-promoting functions, which are vulnerabilities of mutp53 that can be exploited for cancer therapy. Appropriate approaches, such as model organisms, yeast, RNAi, CRISPR, and genetic network screens, are required to make synthetic lethality to p53 mutations a viable option [17]. However, mutp53 acquires oncogenic functions to stimulate multiple signaling pathways to promote tumor progression, which also increases the difficulty of screening potential synthetic lethal partners. Additionally, with increasing studies on the functions of ncRNAs, the importance of exploiting synthetic lethality with mutp53 and ncRNA networks should be mentioned [145]. Wtp53 can regulate miRNA expression via transcriptional or posttranscriptional methods, which supports the theory of screening synthetic lethal partners within miRNAs [108]. While multiple studies have established a link between lncRNAs and circRNAs and mutp53, it should be determined whether the relationships between ncRNAs and mutp53 can be expanded to pan-cancer studies. More studies with compelling evidence focusing on lncRNAs and circRNAs will identify additional synthetic lethality partners of mutp53.

In general, we have a variety of strategies for cancer therapy that target wt/mutp53. Due to the prevalence of p53 mutations in cancer cells, we believe that a more precise classification of mutp53 is required to be consistent with the associated therapeutic strategies. Using mutp53 as an example, p53 LOF mutations can be classified according to their functional alteration, such as deficiency in cell cycle arrest or acquired oncogenic functions to promote glycolysis. Thus, therapeutic targets and relevant agents can be classified differently.

## Conclusions

As an important but highly mutated tumor-suppressive gene, p53 is an attractive therapeutic target for cancer therapy. Reactivating the functions of tumor-suppressive factors is more challenging than directly inhibiting oncogenic factors. Direct targeting of the mutp53 protein is highly dependent on the unique structures of the protein, which makes drug development more complex and limits the applications of drugs. The indirect strategy, which is based on the concept of synthetic lethality, targets the unique vulnerabilities or alterations caused by mutp53 functional defects or acquired oncogenic functions. The synthetic lethality-based approach targeting mutp53, such as utilizing PARP inhibitors to treat cancers harboring BRCA1/2 mutations, can kill tumors with mutp53 while having no or little negative effects on normal cells or tissues. The development of synthetic lethality strategies with mutp53 can bring extensive benefits to patients. Due to the shortcomings in the G1 arrest of TP53-mutated malignancies, current studies focus on the suppression of G2 arrest. However, more studies should be conducted to improve the efficacy of monotherapy, limit the side effects of combination therapies, and expand the repertoire of mutp53 synthetic lethal partners. Based on the research of ncRNAs in cancer, it is reasonable to investigate ncRNAs for synthetic lethality. Although the use of ncRNAs as therapeutic targets for cancer is still in its early stages, it has the potential to expand and fulfil the synthetic lethal network of mutp53. In general, *TP53* is an attractive therapeutic target for cancer, and the development of synthetic lethality with mutp53 will considerably expand clinical options for cancer patients. Furthermore, as more treatments targeting p53 are being developed, it will be feasible to design personalized treatment plans according to the p53 mutation of patients.

## Data Availability

Not applicable.

## Abbreviations

MDM2/X: mouse double minute 2/X
LOFs: loss of functions
GOFs: gain of functions
DBD: DNA-binding domain
wtp53: wild-type p53
mutp53: mutant p53
RS: replication stress
ncRNAs: noncoding RNAs
TCGA: The Cancer Genome Atlas
MDS: myelodysplastic syndromes
CR: complete remission
HDACi: histone deacetylase inhibitor
HDACs: histone deacetylases
CHIP: chaperone-associated ubiquitin ligase carboxy terminus of Hsp70-interacting protein
PDX: patient-derived xenograft
PLK1: polo-like kinase 1
PKMYT1: membrane-associated tyrosine- and threonine-specific cdc2-inhibitory kinase
HNSCC: head and neck squamous cell cancer
OXPHOS: oxidative phosphorylation
GLUT1/4: glucose transporter ¼
SCO2: cytochrome c oxidase 2
AIF: apoptosis-inducing factor
HK2: hexokinase-II
PIP4K2B: PIP kinase genes
PI5P4Kβ: phosphatidylinositol-5-phosphate 4-kinase β
ROS: reactive oxygen species
PFKFB3: 6-phosphofructo-2-kinase/fructose-2,6-biphosphatase 3
EMT: epithelial–mesenchymal transition
ECM: extracellular matrix
PADC: pancreatic ductal adenocarcinoma
PDGFR: platelet-derived growth factor beta
miRNAs: microRNAs
lncRNAs: long noncoding RNAs
PRC: polycomb repressive complex
NF-YA: nuclear transcription factor Y subunit α
SR: serine-/arginine-rich
RBP: RNA-binding protein
ASO: antisense oligonucleotide
